# Optimization of electrode position in electric field treatment for pancreatic cancer

**DOI:** 10.1186/s12876-025-03807-0

**Published:** 2025-04-04

**Authors:** Sangcheol Kim, Yousun Ko, Dongho Shin, Haksoo Kim, Sung Uk Lee, Jonghyun Kim, Tae Hyun Kim, Myonggeun Yoon

**Affiliations:** 1 Department of Biomedical Engineering, Korea University, Seoul, Republic of Korea; 2 Proton Therapy Center, National Cancer Center, Seoul, Republic of Korea; 3 FieldCure Ltd., Seoul, Republic of Korea; 4 School of Biomedical Engineering, Korea University, Seoul, Republic of Korea

**Keywords:** Electric field treatment, Treatment plan, Electrode array, Pancreatic cancer

## Abstract

**Background:**

In electric field-based cancer treatment, the intensity of the electric field applied to the tumor depends on the position of the electrode array, directly affecting the efficacy of treatment. The present study evaluated the effects of changing the position of the electrode array on the efficacy of electric field treatment for pancreatic cancer.

**Methods:**

A 3D model was created based on computed tomography images of 13 pancreatic cancer patients. An electrode array was placed on the surface of the model at various positions, and the electric field was calculated for each. Six treatment plans were created for each patient by rotating each electrode array ± 15⁰, ± 30⁰ in the axial plane, and ± 10⁰ in the sagittal plane relative to the reference plan. The frequency was set at 150 kHz and the current density at 31 mArms/cm^2^ for calculation of all treatment plans. The mean electric field, minimum electric field, homogeneity index (*HI*) and coverage index (*CI*) calculated from the six simulated plans were compared with the reference plan to evaluate the effects of each simulated plan on the tumor.

**Results:**

Comparisons of the simulated plans for each patient with the reference plan showed differences of -2.61 ∼ 11.31% in the mean electric field, -7.03 ∼ 13.87% in the minimum electric field, -64.14 ∼ 13.12% in the *HI*, and − 24.23 ∼ 11.00% in the *CI*. Compared with the reference plan, the optimal plans created by changing the electrode position improved the mean electric field 7.41%, the minimum electric field 7.20%, the *HI* 4.57%, and the *CI* 8.46%.

**Conclusions:**

Use of a treatment planning system to determine the optimal placement of the electrode array based on the anatomical characteristics of each patient can improve the intensity of the electric field applied to the tumor.

## Introduction

Pancreatic cancer is the seventh most common cause of cancer-related deaths in both men and women worldwide and the fourth leading cause of cancer-related deaths in the United States [1, 2]. Estimates indicated that 64,050 persons would be newly diagnosed with pancreatic cancer in the United States in 2023 [1]. Pancreatic ductal adenocarcinoma (PDAC) accounts for approximately 85% of pancreatic malignancies. Although overall survival (OS) has improved significantly in recent years among patients with some types of cancer, OS remains low in patients with PDAC [3]. Due to a lack of effective treatment options, PDAC has been projected to surpass breast cancer as the leading cause of cancer-related deaths in Europe by 2025 [4]. These findings highlight the urgent need for new, innovative treatment methods for pancreatic cancer.

Electric field therapy is a new and alternative cancer treatment modality that targets tumor cells through multiple mechanisms, stopping cell division and ultimately leading to cell death [5]. Electric field therapy has shown clinical benefit for patients with glioblastoma (GBM), suggesting that it may be effective against other cancers. PDAC may be a good candidate for electric field therapy, which is effective for local control, as these tumors tend to spread locally within the abdomen to the liver and peritoneum [6]. In addition, electric field therapy has shown promising results in vitro and in orthotopic tumor models [7, 8]. Moreover, the phase 2 PANOVA-2 trial found that electric field therapy plus gemcitabine (1000 mg/m^2^) or gemcitabine plus nab-paclitaxel (125 mg/m^2^) was safe and well-tolerated in patients with PDAC; based on these preliminary findings, a randomized phase 3 trial (PANOVA-3) is currently in progress [9]. These factors suggest that electric field therapy has potential as a treatment option for pancreatic cancer.

In electric field therapy, an electric field at frequencies ranging from 150 to 200 kHz is applied to an electrode array attached to the surface of a patient’s body [10, 11]. The effectiveness of electric field therapy has been found to be related to the intensity of the electric field applied to the tumor, with a therapeutic threshold of ∼ 1 V/cm [12, 13]. Because the distribution of the electric field in the body can be significantly altered by the placement of the electrode array, optimizing the position of the array is required to increase the intensity of the electric field applied to the tumor. To date, however, few studies have assessed the effect of array placement on the optimization of electric field distribution. In the absence of a 3D treatment planning system, electrode placement-based optimization of the electric field to treat pancreatic cancer is particularly challenging, due to the inhomogeneity of abdominal tissues [14]. The present study used a newly developed 3D treatment planning system to calculate the effects of electrode arrangement on 3D electric field intensity in electric field therapy. The effects of changing the position of the electrode array were evaluated by determining the minimum and mean electric field of the tumor, the homogeneity index (*HI*) and the coverage index (*CI*), parameters commonly used in comparing plans for radiation therapy [15].

## Methods

### Patient data and segmentation

The simulation was based on 3D CT data obtained from 13 pancreatic cancer patients who had undergone radiotherapy at the National Cancer Center Korea (Table 1). Before simulation, the CT data underwent segmentation for bone, lung, heart, esophagus, liver, gallbladder, stomach, pancreas, spleen, kidney, large intestine, small intestine, bladder, muscle and gross tumor volume (GTV), with segmentation confirmed by medical professionals.

**Table 1:** Patient characteristics and treatment settings

Number of patients	Number of males	Number of females	Pathology	Modality	Frequency (kHz)	Current density (mA_rms_/cm^2^)
13	7	6	pancreatic cancer	CT	150	31

### Treatment planning simulation

**Fig. 1:**
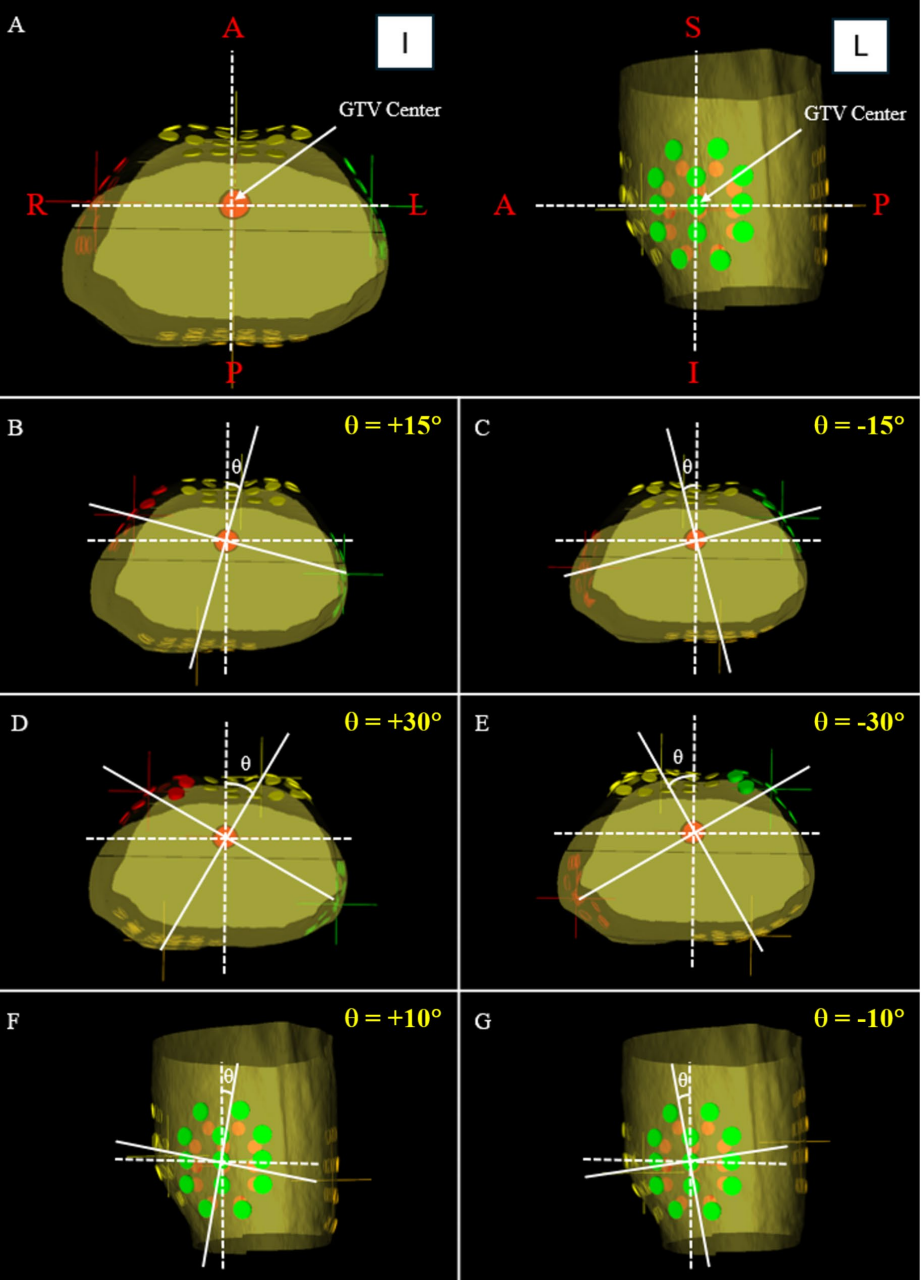
An example of one reference plan and six trial plans. **A** Reference plan. **B** Trial plan rotated θ = +15⁰ in the axial plane. **C** Trial plan rotated θ = -15⁰ in the axial plane. **D** Trial plan rotated θ = +30⁰ in the axial plane. **E** Trial plan rotated θ = -30⁰ in the axial plane. **F** Trial plan rotated θ = +10⁰ in the sagittal plane. **G** Trial plan rotated θ = -10⁰ in the sagittal plane. Here, A, P, L, R, S and I represent anterior, posterior, left, right, superior and inferior, respectively, with angles measured in a clockwise direction defined as positive.

OncoField v1.1.0 (FieldCure, Seoul, ROK) was used for treatment planning simulation of electric field therapy. Based on the study by Rivera et al., the protocol used for plan comparison in this study is outlined as follows [9]. The treatment device generates 150 kHz electric fields in two sequential, perpendicular orientations, delivering a maximum output of 1414 mA RMS (31 mA RMS/cm²) through two pairs of transducer arrays connected to the electric field generator (Table 1). In general, patients were instructed to wear the device for a minimum of 18 h per day to maximize treatment efficacy. Based on clinical trial results, two pairs of electrode arrays, composed of 20 and 13 individual electrodes, were placed in the anterior-posterior and right-left positions, respectively (Fig. 1) [9]. The selection of 20 and 13 electrodes in this study is based on their current clinical use in the treatment of pancreatic and lung cancer [9, 16]. The reference point of the electrode array was set to the center of the tumor, with the two pairs of electrode arrays (anterior to posterior, left to right) created based on the tumor center. This plan was designated the reference plan, with the reference orientation (θ = 0⁰). For comparison, four and two additional trial plans were created by rotating the four electrode arrays by θ = ±15⁰ and θ = ±30⁰ in the axial plane and θ = ±10⁰ in the sagittal plane relative to the reference orientation. Therefore, seven simulation plans were created for each patient by combining one reference plan and six trial plans (Fig. 1).

### Evaluations

For comparison, the minimum and mean intensities of the electric field inside each tumor were calculated for each plan. In addition, based on the simulation results, the homogeneity index (*HI*) and the coverage index (*CI*) were calculated [15]. In radiotherapy, *HI*, an objective tool analyzing the uniformity of dose distribution for the target volume, can be calculated using Eq. (1):

1 $$HI = \frac{{{D_{max}} - {D_{min}}}}{{{D_P}}}$$

where *D*_*max*_, *D*_*min*_ and *D*_*p*_ are the maximum, minimum and prescribed doses, respectively, for the target volume. Because the intensity of the electric field can be regarded as the dose in electric field therapy, *D*_*max*_, *D*_*min*_ and *D*_*p*_ can be defined as the maximum, minimum and prescribed intensities, respectively, of the electric field. The prescribed dose, *D*_*p*_, was set at the mean intensity of the electric field for the reference plan. By definition, a lower *HI* is indicative of a more homogeneous target dose.

The *CI*, an objective tool to analyze the coverage of the target volume by the prescribed dose, can be calculated using Eq. (2):

2 $$CI = \frac{{{V_{100}}_{GTV}}}{{{V_{GTV}}}}$$

where *V*_*100GTV*_ represents the volume receiving at least 100% of *D*_*p*_ within the GTV, and *V*_*GTV*_ represents the volume of the entire GTV. Therefore, a *CI* closer to 1 is indicative of better coverage of the target volume by the prescribed dose.

For comparison, *HI* and *CI* were normalized to the values of the reference plan. The plan with the lowest values of *HI* was selected as the best plan, whereas the plan with the highest minimum, mean intensities of the electric field and *CI* was selected as the best plan. In addition to these evaluation metrics, plans were qualitatively compared based on their dose-volume histograms (DVH), a plot of the volume of a given structure receiving a certain dose or higher as a function of dose [17]. DVH, a histogram relating dose to tissue volume, is generally used when comparing competing treatment plans in radiotherapy.

## Results

**Fig. 2:**
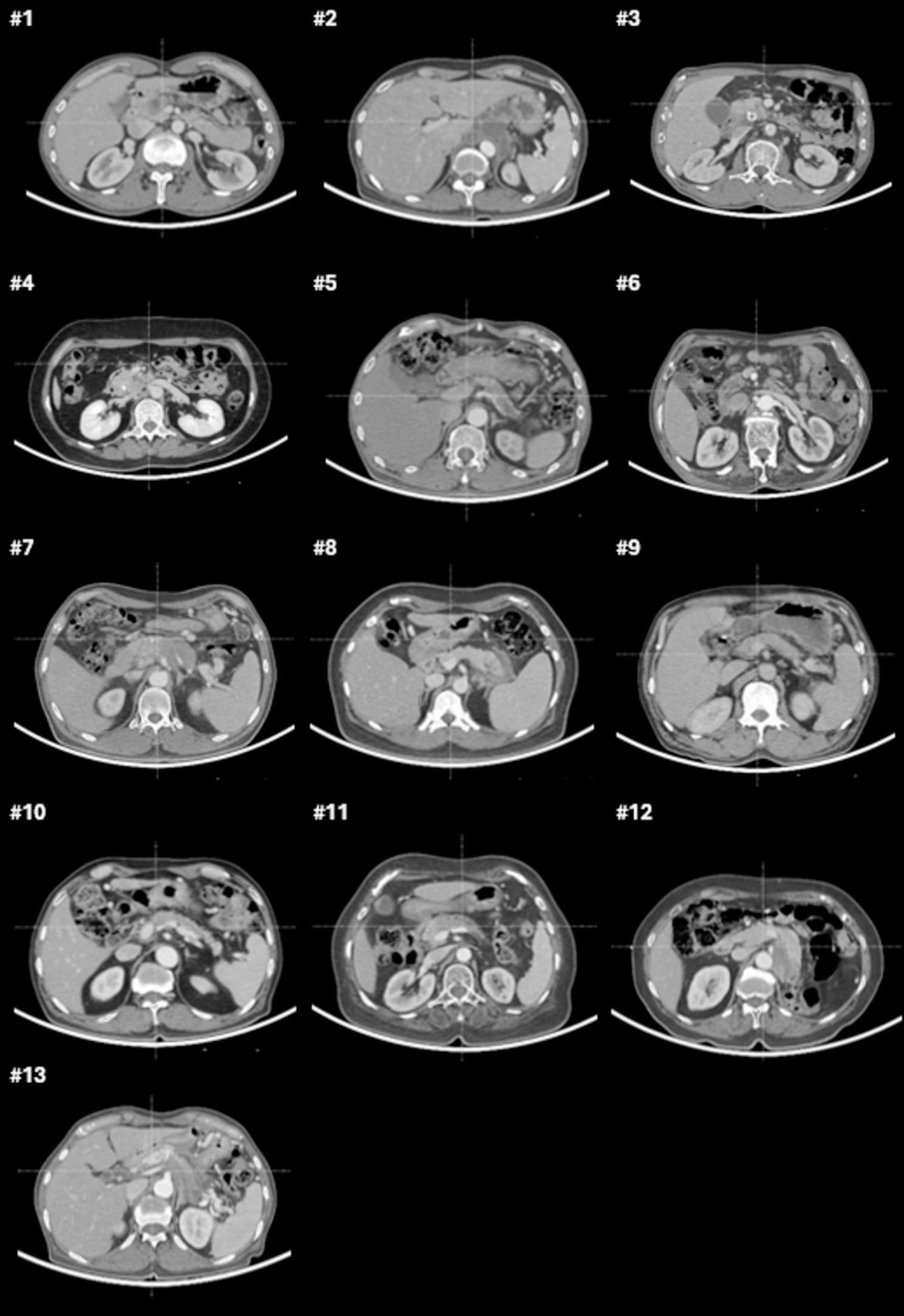
Axial CT slice images taken at the reference point are provided for all 13 patients, offering a visual representation of the anatomical structures and electrode placements used in the study.

Figure 2 shows axial CT slice images at the center of the tumor for all 13 patients. Table 2 compares various indices of the reference plan with the indices of the six trial plans. The best of the six trial plans was selected based on previously described criteria. The values in Table 2 represent the percentage improvement of indices of the best trial plan compared with the reference plan. Comparisons of mean and minimum electric field intensities showed that the trial plans outperformed the reference plans in all 13 patients. Similarly, comparisons of *CI* showed that the trial plans performed better than the reference plans in all patients. Evaluation of *HI* showed that the trial plans were equal to or greater than the reference plan in all 13 patients.

**Table 2:** Percentage improvement of the best trial plan compared with the reference plan in the 13 patients with PDAC

Variables (%)	1	2	3	4	5	6	7	8	9	10	11	12	13
Mean Intensity	8.61	8.00	9.26	10.98	6.06	3.24	7.73	11.31	5.64	9.00	4.95	5.10	6.50
Minimum Intensity	9.42	8.54	7.30	7.50	8.20	5.41	9.84	5.74	10.53	13.87	3.18	2.84	1.19
*HI*	9.29	4.94	0.00	7.71	9.59	0.00	4.10	2.73	0.00	4.92	13.12	0.00	3.03
*CI*	10.63	11.00	9.08	8.84	9.83	4.68	8.89	10.42	9.43	8.98	9.39	4.89	3.87

**Fig. 3:**
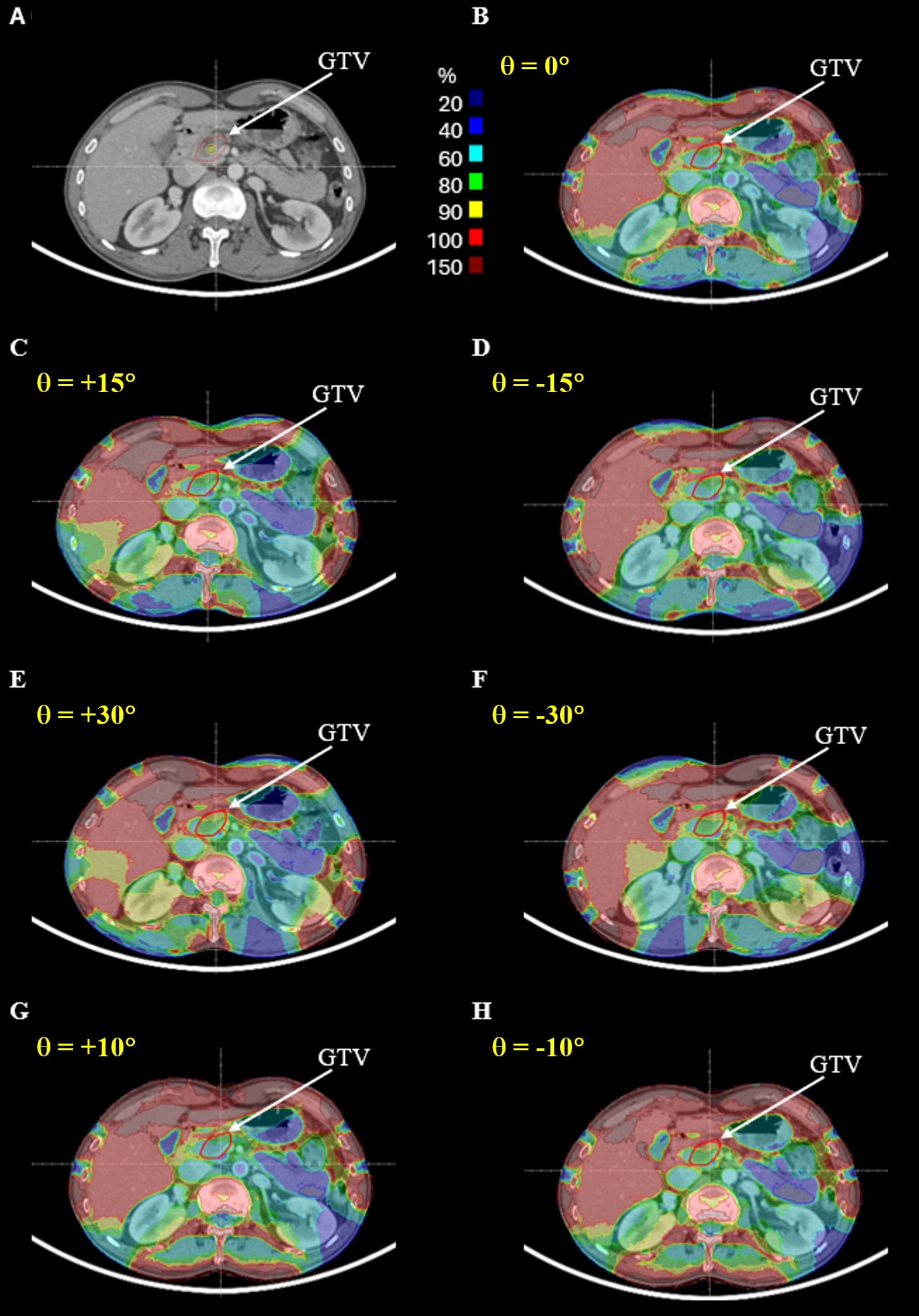
Dependence of electric field distribution on electrode locations for representative patient 1. **A** Axial CT image at the center of the tumor. **B** Reference plan. **C** Trial plan rotated θ = +15⁰ in the axial plane. **D** Trial plan rotated θ = -15⁰ in the axial plane. **E** Trial plan rotated θ = +30⁰ in the axial plane. **F** Trial plan rotated θ = -30⁰ in the axial plane. **G** Trial plan rotated θ = +10⁰ in the sagittal plane. **H** Trial plan rotated θ = -10⁰ in the sagittal plane.

**Fig. 4:**
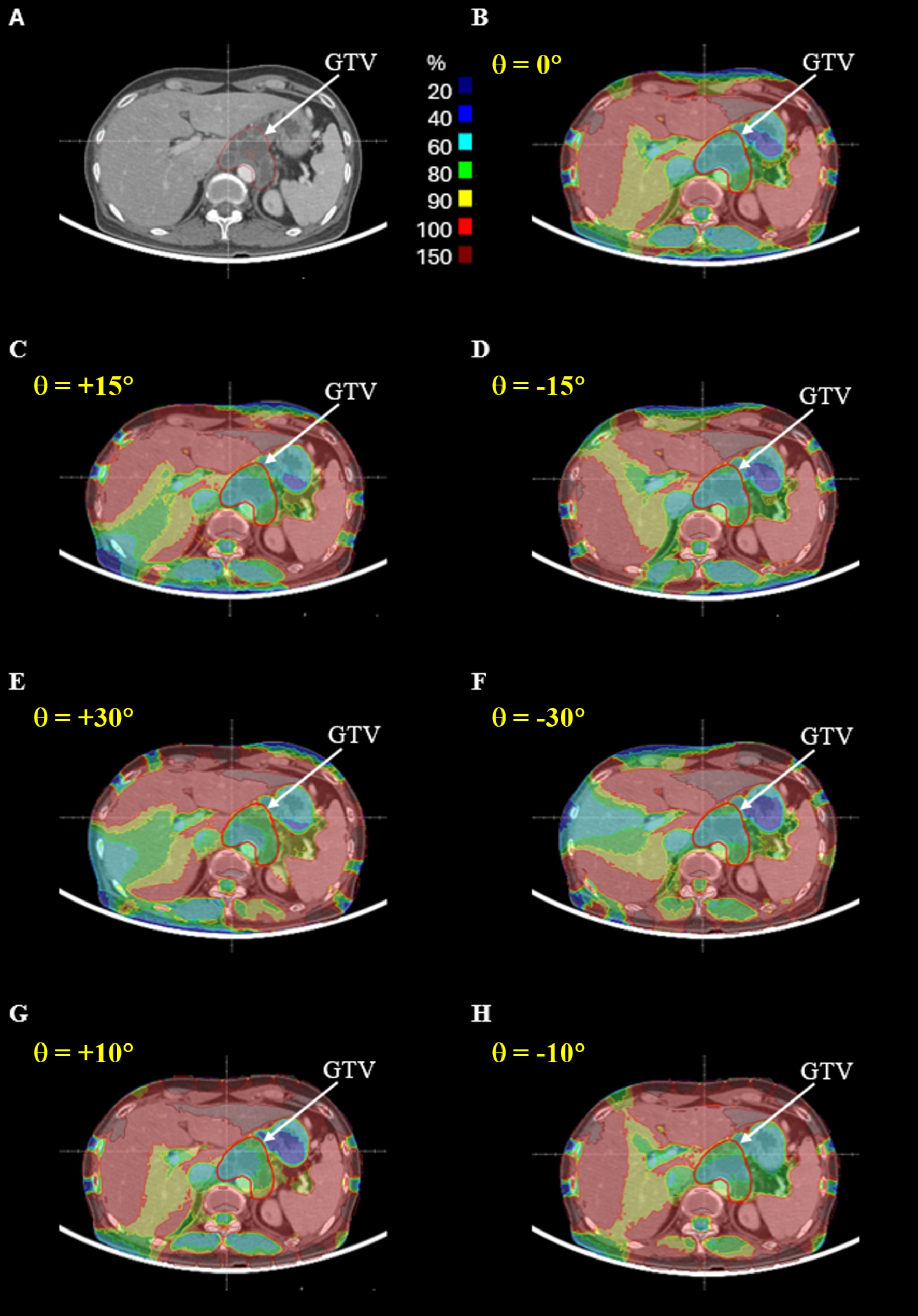
Dependence of electric field distribution on electrode locations for representative patient 2. **A** Axial CT image at the center of the tumor. **B** Reference plan. **C** Trial plan rotated θ = +15⁰ in the axial plane. **D** Trial plan rotated θ = -15⁰ in the axial plane. **E** Trial plan rotated θ = +30⁰ in the axial plane. **F** Trial plan rotated θ = -30⁰ in the axial plane. **G** Trial plan rotated θ = +10⁰ in the sagittal plane. **H** Trial plan rotated θ = -10⁰ in the sagittal plane.

**Fig. 5:**
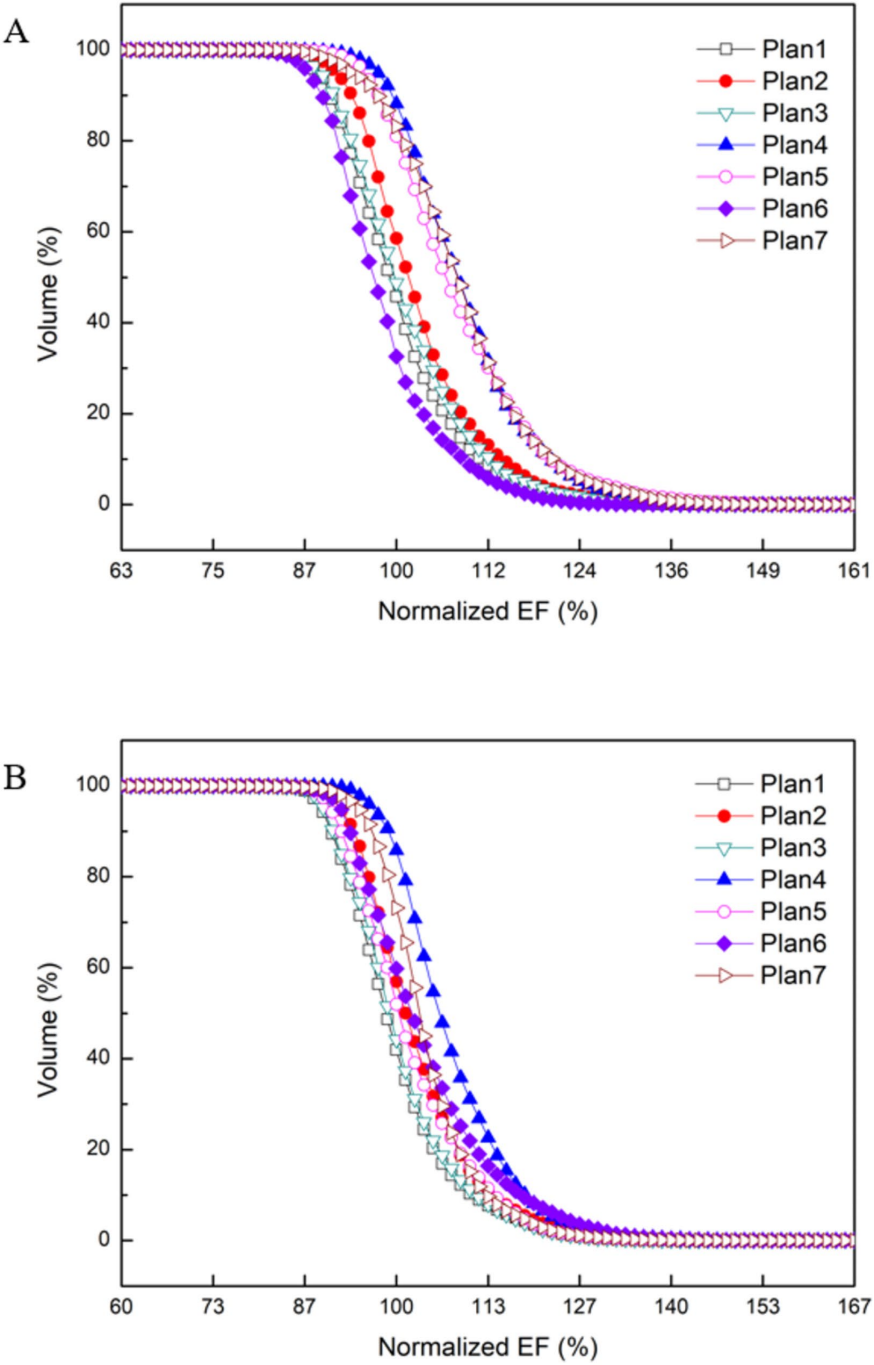
**A** Dose volume histograms (DVHs) of representative patient (1). **B** DVHs of representative patient (2). Here, normalized EF means the normalized electric field in which 100% is the intensity of the mean electric field inside the tumor for the reference plan.

Figures 3 and 4 show the electric field intensity distributions for the first and second representative patients, respectively, across the different trial plans. These plots show that the electric field intensity within each tumor was dependent on the positions of the electrodes. Figure 5 shows the DVH for the GTV of these two representative patients. Plan1 indicates the reference plan; Plan2, Plan3, Plan4 and Plan5 represent trial plans rotated by θ = +15⁰, -15⁰, + 30⁰, and − 30⁰, respectively, in the axial plane; and Plan6 and Plan7 represent trial plans rotated by θ = +10⁰ and − 10⁰, respectively, in the sagittal plane.

These results indicated that the intensity of the electric field inside the body of the patient depended significantly on the electrode position. Compared with the reference plan for the first representative patient, plan 4, which was rotated + 30⁰ in the axial plane, showed the best results, including optimal values for minimum and mean electric field intensity and *CI*. In contrast, plan 6, which was rotated + 10⁰ in the axial plane, showed the poorest results, with the worst values for minimum and mean electric field intensity and *CI*. Of the six trial plans, five showed better values for minimum and mean electric field intensity and *CI* than the reference plan (Fig. 5A).

Similar results were observed for the second representative patient, with plan 4, which was rotated + 30⁰ in the axial plane, showing the best result, with optimal values for minimum and mean electric field intensity and *CI*. In this patient, all six trial plans showed better values for minimum and mean electric field intensity and for *CI* than the reference plan (Fig. 5B). Unlike the results of the first representative patient, there were no large differences between the trial plans and the reference plan for the second representative patient. These results suggest that an optimized plan based on electrode placement can provide a higher intensity electric field to the tumor than the reference plan.

Table 3 shows the mean percentage improvement of the best plan for each index compared with the reference plan. Compared with the reference plan, the mean field intensity and minimum field intensity were improved by 7.41% and 7.20%, respectively, whereas the *HI* and *CI* of the optimized plan were improved by 4.57% and 8.46%, respectively. These findings confirmed that the plans with the optimal electrode array position could produce better results than the reference plan, in which electrode arrays are attached at a reference position, irrespective of the electric field intensity in the tumor.

**Table 3:** Percentage improvement of the best trial plan for various indices compared with the reference plan

	Mean intensity of electric field	Minimum intensity of electric field	HI	CI
Mean Improvement(%)	7.41	7.20	4.57	8.46

## Discussion

This study evaluated the optimal electric field treatment plan based on the positioning of an electrode array in patients with pancreatic cancer. Compared with the reference plan, the optimal plans, created by changing the position of the electrode array, improved the mean and minimum intensity of the electric field by 7.41% and 7.20%, respectively, as well as improving *HI* and *CI* by 4.57% and 8.46%, respectively. In addition, the intensity of the electric field inside the tumor was found to depend greatly on the position of the electrode array, and to be affected by the location of organs and tumors within each patient’s body.

Because the pancreas is located close to the center of the body, the pairs of electrode arrays of the reference plan were determined to be attached in the anterior-posterior and left-right directions relative to the center of the tumor. Because the effectiveness of electric field therapy was reported to be generally proportional to the intensity of the electric field applied to the tumor [12, 13], the mean electric field, minimum electric field, *HI* and *CI* of the six trial plans were compared with those of the reference plan. In general, higher mean and minimum electric field and higher *CI* are indicative of a higher intensity electric field inside the tumor, enhancing the efficiency of electric field therapy. The fourth parameter, *HI*, was an indicator of the uniformity of the electric field applied within the tumor.

A previous analysis of the effects of electrode positioning on electric field intensity in the treatment of brain cancers showed that, depending on the position of the two pairs of electrode arrays, the electric field intensities of the optimized plan were 10 ∼ 17% higher than those of the reference plan [18]. Similarly, the present study found that the optimized plan, in which the position of the electrode array was altered from that in the reference plan, resulted in a higher electric field inside the tumor compared with the reference plan. Evaluation of the electric field intensity inside the tumor showed that the plans in which each electrode array was rotated + 30⁰ and − 30⁰ in the axial plane yielded the highest electric field intensities inside the tumor for three and six patients, respectively. In contrast, the plan in which each electrode array was rotated − 10⁰ in the sagittal plane yielded the highest electric field intensity inside the tumor for the remaining four patients. Because the electric field distribution is significantly affected by the location, shape and size of the tumor, as well as those of various organs, it is difficult to predict the optimal location of the electrode array without actually calculating the electric field distribution inside the body of each patient using a 3D treatment planning system.

While electric field therapy is generally well tolerated, it can cause certain side effects. Since the treatment requires adhesive electrode arrays to be placed on the skin near the tumor site, most side effects are localized to these areas. One of the most commonly reported side effects is skin irritation and dermatitis, which may present as redness (erythema), rash, itching (pruritus), and, in some cases, blistering or ulceration. Additionally, because electric field therapy requires patients to wear the device for at least 18 h a day, prolonged use may contribute to physical exhaustion over time. To date, there is no strong evidence indicating that electric field therapy cause organ toxicity, such as damage to the lungs, heart, liver, or kidneys, provided that the therapy operates within a safe temperature range. However, it remains important to minimize the intensity of the electric field in normal tissues, as excessive exposure could potentially generate heat or cause unintended damage to internal structures.

The clinical implementation of electric field therapy requires expertise from oncologists, medical physicists, nurses, and device specialists to ensure proper treatment planning, verification, and patient adherence. Verification methods, such as array placement monitoring, adherence tracking, and skin condition assessment, play a crucial role in optimizing treatment delivery. However, variations in patient adherence, electrode positioning, and tumor progression can lead to differences between the planned and delivered treatment. Therefore, regular monitoring and patient support are essential for maximizing treatment effectiveness while minimizing side effects. In addition, ensuring the accurate delivery of the intended electric field during treatment is a crucial aspect of clinical implementation. Yoon et al. investigated a quality assurance (QA) system for electric field therapy using a water phantom, enabling verification of three-dimensional E-field distributions [19]. Their study found that in 3D evaluations, the average differences in voltage and electric field were 1.06% and 6.65%, respectively. These findings are expected to enhance the quality of electric field therapy by providing experimental validation of electric fields and improving predictions of treatment efficacy. However, further research is necessary, particularly on inhomogeneous phantoms, such as solid phantoms, which could aid in developing humanoid models. These advanced models would enable more precise quality assurance (QA), allowing for a more accurate simulation of realistic tissue conditions and further improving treatment accuracy.

While this study provides valuable insights into the efficacy and feasibility of the treatment planning system for electric field therapy, several limitations must be acknowledged. First, the small sample size restricts the ability to draw definitive conclusions, limiting statistical power and generalizability. Expanding the sample size in future studies will be essential to enhance statistical robustness, improve the applicability of results across diverse patient populations, and minimize potential biases. Second, this study focuses on optimizing electrode positions based on electric field intensity to enhance treatment effectiveness. However, a key limitation is the lack of direct clinical outcome data, such as tumor response, patient survival, or treatment-related adverse effects. While optimizing electrode placement is essential for maximizing field distribution, the actual therapeutic impact on patients remains to be validated through clinical research. Future studies should bridge this gap by combining computational optimization with clinical outcome analysis to demonstrate the real-world benefits of optimized electric field therapy. Third, while this study optimizes electrode positions based on electric field intensity, alternative optimization strategies, such as conformal electric field application or intensity-modulated electric field application based on voltage variation, may offer further improvements in treatment efficacy. These methods could enable more precise and patient-specific electric field distributions, potentially enhancing tumor targeting while minimizing exposure to surrounding healthy tissues. Future studies should explore these advanced strategies to evaluate their feasibility, therapeutic benefits, and clinical impact.

Additionally, the effectiveness of electric field therapy varies due to patient-specific factors, electrode positioning variations, and anatomical changes during treatment. Differences in electrical conductivity, tissue density, and tumor composition influence field distribution, with water-rich tissues (e.g., muscles, tumors) conducting fields more efficiently than fat or bone. Variability in tumor response and skin sensitivity further affects treatment outcomes. Personalized treatment planning and computational modeling can help optimize therapy for each patient. Regular monitoring and patient education are crucial for maintaining consistent array positioning and adherence. Furthermore, anatomical changes during treatment, such as tumor shrinkage, progression, or weight fluctuations, can alter field distribution, while surgery, radiation, or fluid retention may further affect field penetration. Routine imaging and adaptive electrode placement are necessary to maintain optimal treatment delivery.

In summary, patient-specific factors, positioning accuracy, and anatomical changes significantly influence electric field therapy. Regular monitoring, imaging assessments, and treatment adjustments are essential to maximize treatment efficacy and improve clinical outcomes. Future studies should focus on expanding sample sizes, integrating clinical outcome data, and exploring advanced optimization techniques to further refine electric field therapy and enhance its real-world applicability.

## Conclusions

The present study assessed the impact of adjusting electrode array positions during electric field treatment of pancreatic cancer. The optimized plan was found to improve the mean electric field, minimum electric field, *HI*, and *CI* compared with the reference plan. Optimizing electrode placement may increase electric field intensity within the tumor, potentially enhancing treatment efficacy.

## Data Availability

No datasets were generated or analysed during the current study.

## Abbreviations

HI: Homogeneity Index
CI: Coverage Index
GTV: Gross Tumor Volume
DVH: Dose-Volume Histogram
